# Genotypic Distribution of *FGF4L2* in DTK-Registered Dachshunds in Germany: A Pilot Study

**DOI:** 10.3390/genes17080969

**Published:** 2026-08-19

**Authors:** Hanna Berls, Jan Peter Bach, Danika Bannasch, Peter J. Dickinson, Holger A. Volk

**Affiliations:** 1 Department of Small Animal Medicine and Surgery, University of Veterinary Medicine Hannover, 30559 Hannover, Germany; hanna.berls@tiho-hannover.de; 2 German Kennel Club (Verband für das Deutsche Hundewesen, VDH) e.V., 44141 Dortmund, Germany; bach@vdh.de; 3 Department of Population Health and Reproduction, University of California-Davis, Davis, CA 95616, USA; dlbannasch@ucdavis.edu; 4 Department of Surgical and Radiological Sciences, University of California-Davis, Davis, CA 95616, USA; pjdickinson@ucdavis.edu

**Keywords:** *FGF4L2* retrogene, Dachshund, allele frequency, chondrodystrophy, intervertebral disc extrusion, genetic screening

## Abstract

Background/Objectives: The fibroblast-growth-factor 4 retrogene insertion on chromosome 12 (*FGF4L2*) is known to be associated with chondrodystrophy and intervertebral disc disease (IVDD), which increases the risk of Hansen’s type I intervertebral disc extrusion (IVDE) in Dachshunds. While the *FGF4L2* insertion is known to occur at high frequency within the breed, subgroup-specific data relating to coat-type and size categories remain limited. This pilot study aimed to determine the distribution of *FGF4L2* and wild-type (*N*) alleles in a cohort of Dachshunds registered with the German Dachshund Club (DTK) and to evaluate differences among coat and size varieties. Methods: A total of 488 Dachshund samples from the DNA archive of the Deutscher Teckelklub 1888 e.V. (DTK) were analysed and genotyped. Genotype data was categorised according to coat-type and size variant. Allele and genotype frequencies were calculated for the overall population and for each subgroup. Results: The overall frequency of the *FGF4L2* insertion allele was 96.51% (95% CI: 95.17–97.50). However, there were moderate differences between subgroups. The allele was nearly fixed in several coat and size variants. Standard wire-haired Dachshunds had the lowest allele frequency (89.09%) and were the only group in which homozygous wild-type individuals were observed. Heterozygous frequencies peaked in standard smooth-haired (16.7%) and standard wire-haired (14.5%) groups. Conclusions: Because the *FGF4L2* frequency differs meaningfully between Dachshund varieties, grouping them into a single breed, as many studies do, can obscure the residual subgroup-specific genetic variation present in this population. Analysing the varieties separately provides a basis for future studies investigating factors that may interact with *FGF4L2* in IVDE, thereby highlighting its multifactorial nature. It also reveals where wild-type alleles persist, making genotype-informed, variety-specific breeding a viable strategy for improving vertebral column health. However, variation in *FGF4L2* allele frequency alone may not fully account for the recently reported differences in vertebral column health between Dachshund coat varieties.

## 1. Introduction

Chondrodystrophic dog breeds are characterised by premature degeneration of the intervertebral discs (IVDs), which predisposes them to Hansen’s type I intervertebral disc extrusion (IVDE) [1]. IVDE often results in compression of the spinal cord and nerve roots, which can lead to pain and neurological deficits such as paresis and paralysis, requiring frequently surgical intervention [1,2,3,4]. Dachshunds are considered one of the breeds at highest risk for clinically apparent IVDE [5,6], with approximately 16–25% reported to develop clinically apparent disease during their lifetime [7,8,9]. Accordingly, Dachshunds have been estimated to be 10–12 times more likely than many other dog breed to develop clinically apparent IVDE [7].

Thus, the distinctive skeletal phenotype of Dachshunds and other chondrodystrophic breeds is associated with marked predisposition to Hansen type I disc extrusion [1]. The link between acute disc extrusion and chondrodystrophy in predisposed breeds like Pekingese, French Bulldogs, and Dachshunds was initially reported by Hansen [1]. These disc extrusions typically occur in the thoracolumbar region of the spine and may lead to severe neurological injury [1,5].

Previous genetic research has shown that chondrodystrophy is caused by the insertion of the *fibroblast-growth-factor 4* retrogene on chromosome 12 (*FGF4L2*), which is also responsible for premature disc degeneration in dogs carrying this insertion [10,11]. *FGF4* is essential for organogenesis and embryonic development [12], particularly in later stages when *FGF4* is highly expressed in the apical ectodermal ridge of the developing limb bud and somites, which later form the vertebral column and non-nuclear components of the intervertebral disc [13,14,15].

Two retrogenes that arise from retrotransposition in dogs, in addition to the parental *FGF4* gene on chromosome 18, are considered to be the origin of varying degrees of disproportionate dwarfism and skeletal dysplasia [10,16]. Retrotransposition develops an intron-free duplicate of the original gene by converting a processed mRNA molecule back into DNA and integrating it somewhere else in the genome [17,18].

The first *FGF4* retrogene, located on chromosome 18 (*FGF4L1*, previously CFA18-FGF4, CDPA), is primarily associated with chondrodysplasia and pronounced shortening of the limbs [16], while the second retrogene on chromosome 12 (*FGF4L2*, previously CFA12-FGF4, CDDY) has been implicated in chondrodystrophy and is associated with milder limb length variation [19] but, as aforementioned, is also associated with premature degeneration of IVDs and a predisposition to clinically apparent disease secondary to extrusion of the degenerative discs [10]. In the Dachshund population, both retrogene allele insertions are nearly fixed, which gives rise to their characteristic phenotype [17].

While the *FGF4L2* insertion is widespread throughout the canine population, occurring at an allele frequency of approximately 11–14% in more than one million dogs and being present in 89 breeds or breed varieties at frequencies ≥ 1% [20], it is nearly fixed in Dachshunds and several other breeds like beagles or cocker spaniels.

Recently, significant differences in vertebral column health between the Dachshund coat and size varieties were described across three European populations. Smooth-coated Dachshunds consistently showed poorer vertebral column health than wire- and long-haired Dachshunds, whereas the influence of body size was less consistent. These findings highlight clinically relevant differences between Dachshund coat and size varieties and support analysing these subgroups separately in future genetic studies [21].

Against this background, *FGF4L2* is of particular importance in the context of Dachshund breeding, as breed-related health issues have recently received increasing attention. In Germany, debates concerning “Qualzuchten,” or torture breeding—breeding that results in pain, suffering, or injury—have brought attention to the need to balance breed-defining traits with animal welfare considerations. The *FGF4L2* insertion has been associated with premature degeneration of the intervertebral discs, which increases the likelihood of spinal cord compression, and the development of clinically apparent IVDE with pain and neurological deficits.

Given this increased risk of intervertebral disc disease, the genetic contribution of the *FGF4L2* insertion, and the emerging evidence of differences between Dachshund coat and size varieties, knowledge of subgroup-specific allele frequencies within breeding populations is essential for informed and health-oriented breeding decisions. However, data on allele frequencies in German Dachshunds registered with the DTK are currently lacking.

The aim of this pilot study was to generate representative data of *FGF4L2* and wild-type allele frequencies in DTK-registered Dachshunds in Germany, across the nine coat and size varieties. Our hypothesis was that the allele insertion of *FGF4L2* is nearly fixed in the population, but would occur at a lower frequency in the wire-haired standard Dachshunds, reflecting the lower reported prevalence of clinically apparent IVDE in this subgroup [7].

## 2. Materials and Methods

### 2.1. Study Population

This study was designed as a pilot allele frequency study in Dachshunds in Germany. A total of 488 dogs were included. Samples were obtained from the DNA archive of the German breed club “Deutscher Teckelklub (DTK)”, where blood samples have been routinely collected since 2012 as part of the mandatory genetic screening programme for breeding approval. In 2024, these archived samples were used to initiate the present study. All samples were archived and stored under standardised conditions.

In Germany and other countries following the FCI standard, Dachshunds are divided into three size varieties based on chest circumference: standard Dachshund, miniature Dachshund, and rabbit Dachshund. In addition to size varieties, Dachshunds can be categorized into three coat types: smooth-haired, long-haired, and wire-haired. The combination of these size and coat varieties results in a total of nine distinct Dachshund groups.

The study population consisted exclusively of DTK-registered Dachshunds. Within the DTK breeding programme, coat-type and size varieties are maintained as separate breeding populations, with pedigree information routinely verified through DNA-based parentage testing for more than 15 years. The study population comprised Dachshunds of the different coat types and sizes. Approximately equal numbers of samples were available for each of the 9 subgroups, with 53–55 dogs per coat-type and size category. Samples were randomly selected from the archived material to ensure balanced representation of the different Dachshund varieties.

### 2.2. DNA Samples

DNA templates were obtained from canine blood samples stored on GOcards^®^ (Generatio GmbH, Heidelberg, Germany), archived as part of the DNA programme of the DTK. Using a sterile punch, discs of 1 mm diameter were excised from the blood cards. Each disc was washed according to the manufacturer’s instructions to remove potential PCR inhibitors and was subsequently used directly as template material in PCR reactions.

### 2.3. PCR Assay and Genotyping

Detection of the *FGF4L2* retrogene insertion was performed using a previously described three-primer PCR assay. Primer design and amplification conditions were based on the protocol reported by Embersics et al. [22], with the modification of a FAM-labelled external reverse primer (Biomers, Ulm, Germany) to enable fluorescent fragment detection.

PCR products were separated by capillary electrophoresis using an ABI 3130xl Genetic Analyzer (Applied Biosystems, Foster City, CA, USA). Electropherograms were analysed to determine fragment sizes, and genotypes were assigned based on the presence or absence of insertion-specific amplicons as described by Embersics et al. [22].

### 2.4. Statistical Analysis

Genotype frequencies were defined as the proportions of dogs with the *FGF4L2/FGF4L2*, *N/FGF4L2*, and *N/N* genotypes within the study population or subgroup. Heterozygous frequency was calculated as the proportion of heterozygous dogs among all tested individuals within the respective population or subgroup.

Allele frequencies and corresponding 95% Wilson confidence intervals were calculated. Differences in genotype distributions among the nine Dachshund varieties were assessed using Fisher’s exact test on a 9 × 3 contingency table. Hardy–Weinberg equilibrium was assessed using the exact test implemented in the HardyWeinberg package (v. 1.7.9) for R for both the pooled dataset and each Dachshund variety separately. A two-sided *p*-value < 0.05 was considered statistically significant. All statistical analyses were performed in R version 4.6.1 [23]. Figures were generated using the ggplot2 package (v. 4.0.3).

### 2.5. Ethics Statement

Samples were collected with owner consent by the Deutscher Teckelklub (DTK) as part of its routine genetic screening programme for DTK-registered Dachshunds, in which DNA samples have been routinely collected from all puppies registered with the DTK since 2012, and stored in a genetic database. The samples analysed in this study were obtained from this existing DNA archive and were not collected specifically for research purposes, and no additional procedures were performed for the study. The project was registered as part of a doctoral research programme and underwent review by the local ethics and animal welfare committee.

As the study exclusively analysed previously collected samples obtained as part of the routine genetic screening programme for DTK-registered Dachshunds, no additional ethical approval was required under the local regulations of the University of Veterinary Medicine Hannover Foundation and Lower Saxony.

## 3. Results

A total of 488 Dachshunds were genotyped for the *FGF4L2* retrogene insertion. Of these, 456 Dachshunds (93.44%) were homozygous for the *FGF4L2* retrogene insertion. Thirty Dachshunds (6.15%) were heterozygous and two (0.41%) were homozygous for the wild-type *N/N* genotype (Table 1).

The overall insertion allele frequency in the study population was 96.51% (95% CI: 95.17–97.50). The wild-type allele frequency was 3.49% with only 6.15% heterozygous Dachshunds.

**Figure 1:**
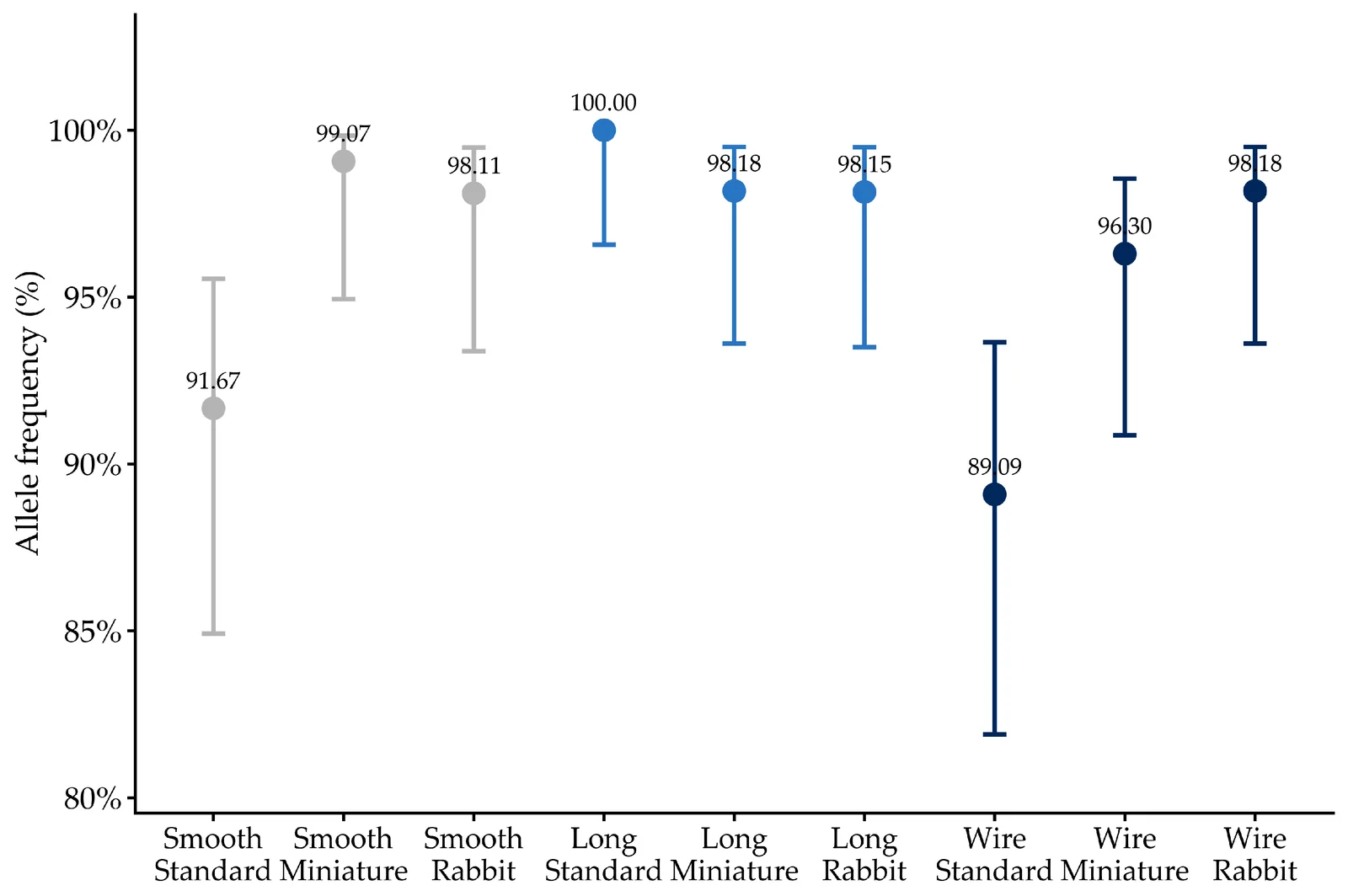
*FGF4L2* allele frequencies (%) across the nine Dachshund varieties. Points represent allele frequencies, and error bars indicate the corresponding 95% Wilson confidence intervals. Exact allele frequencies are displayed above each estimate.

Furthermore, the allele frequencies of *FGF4L2* were evaluated in relation to different coat types and varieties of Dachshund size (Table 2, Figure 1). The corresponding genotype distributions for each subgroup are presented in Supplementary Figure S1. These distributions differed significantly among the nine Dachshund varieties (Fisher’s exact test, *p* = 0.0012). The allele frequencies in the subgroups ranged from 89.09% in the wire-haired standard group to 100% in the long-haired standard group.

The allelic frequencies of *FGF4L2* in the wire-haired rabbit Dachshund group, in the smooth miniature and smooth rabbit Dachshund groups, and in all three long-haired sizes were >98%. The wire-haired miniature group had a frequency of 96.3%, and the smooth standard Dachshunds had a frequency of 91.67% (Table 2).

The lowest allele frequency in this pilot study was found in the wire-haired standard Dachshund subgroup, with an allele frequency of 89.09% (Table 2). This subgroup comprises the only wild-type genotypes (*N/N*) in two standard wire-haired Dachshunds in the entire dataset.

Additionally, this group comprised eight Dachshunds with a heterozygous *N/FGF4L2* genotype, representing a heterozygous frequency of ≈14.5%.

The group with the highest heterozygous frequency was the smooth-haired standard group with ≈16.7% (9/54 Dachshunds).

Genotype frequencies did not significantly deviate from Hardy–Weinberg equilibrium in the pooled dataset (exact test, *p* = 0.109). Likewise, no significant deviations from Hardy–Weinberg equilibrium were detected in any of the nine Dachshund varieties (all exact test *p* ≥ 0.106; Supplementary Table S1).

## 4. Discussion

This pilot study aimed to generate representative data on the frequencies of the *FGF4L2* and wild-type alleles in German Dachshunds registered with the DTK, and to define the distribution of different genotypes among various coat and size types.

To the authors’ knowledge, this is the first study to investigate *FGF4L2* allele frequencies across all nine Dachshund coat-type and size varieties represented within the German DTK population. The *FGF4L2* insertion allele was found in 96.51% (95% CI: 95.17–97.50) of Dachshunds, but there were some differences between coat and size variants. It was almost fixed in several coat and size variants. Standard wire-haired Dachshunds had the lowest allele frequency (89.09%) and were the only group in which homozygous wild-type individuals were observed. Heterozygous frequencies were highest in the standard smooth-haired (16.7%) and standard wire-haired (14.5%) groups.

Although the overall frequency of the *FGF4L2* insertion allele is high, which is consistent with the known genetic characteristics of the breed [11,17], the most notable finding of this study was the variation observed among the different coat and size subgroups.

In particular, the *FGF4L2* allele appeared to be nearly fixed in some coat and size types, whereas a lower frequency and the only wild-type genotypes were identified in wire-haired standard Dachshunds. These results could indicate a degree of genetic differentiation among Dachshund subgroups, potentially reflecting their separate breeding histories.

A recently published study demonstrated significant differences in spinal health between Dachshund coat and size varieties across three European populations, with smooth-coated Dachshunds consistently showing poorer vertebral column health than wire- and long-haired Dachshunds [21]. These findings further support the concept that Dachshund subgroups should not be considered as a homogeneous population when investigating genetic factors associated with IVDE. While the extent to which these subgroup differences are explained by variation in *FGF4L2* allele frequency remains unclear, our results provide the first subgroup-specific *FGF4L2* allele frequency data for DTK-registered Dachshunds and establish a baseline for future studies investigating the genetic basis of subgroup-specific differences in vertebral column health.

The observed subgroup differences should be interpreted in the context of the structured breeding system of the DTK population. In practice, the different coat varieties are bred separately within the DTK breeding programme, whereas matings between adjacent size varieties (rabbit Dachshund × miniature Dachshund and miniature Dachshund × standard Dachshund) are generally permitted.

The relatively higher heterozygous frequencies observed in standard smooth-haired and standard wire-haired Dachshunds may be relevant for future breeding strategies, as these subgroups retain greater genetic variation at the *FGF4L2* locus.

Additionally, genotype distribution did not deviate significantly from Hardy–Weinberg equilibrium.

To better contextualise these findings, it is important to consider the biological role of the *FGF4* retrogenes and the historical breeding background of the Dachshund.

The high overall frequency of the *FGF4L2* retrogene insertion allele likely reflects ongoing selection associated with breed-defining phenotypic traits over the centuries. There are two common FGF4 retrogene insertions in the canine population: *FGF4L1* and *FGF4L2* [10,16]. The *FGF4L1* retrogene insertion on chromosome 18 is associated with chondrodysplasia and limb shortening [16,19], providing a major aspect of the specific Dachshund appearance, whereas *FGF4L2* has a lesser influence on limb shortening but contributes significantly to the characteristic chondrodystrophic phenotype and is associated with intervertebral disc extrusion [10,19]. From a historical viewpoint, Dachshunds were selectively bred for hunting and underground work, favouring a characteristic body conformation with shortened limbs and an elongated body [24], which may have led to continuous selection towards an increasing number of both retrogenes. As a consequence, the low frequency of wild-type alleles observed in many Dachshund subgroups may reflect long-term selection for the characteristic chondrodystrophic phenotype.

However, beyond this overall pattern, the marked differences between subgroups indicate that allele distribution is also shaped by subgroup-specific breeding histories. The marked differences in allele frequencies between Dachshund subgroups may reflect a combination of separate breeding histories, historical selection, and the breeding structure of the individual varieties. Further studies have shown that geographical isolation and also selective breeding techniques can result in genetically distinct subpopulations developing within dog breeds, thereby decreasing gene flow between lineages [25]. Divided by size and hair type, the subgroups are essentially separate breeding populations within the breed, leading to divergent allele frequencies and minimal genetic exchange. Additionally, breeding objectives differed between Dachshund subgroups, which may have contributed to differences in allele frequencies.

To place these findings into context, it is important to compare them with previously published data on *FGF4L2* allele frequencies. Between 2019 and 2025, previous studies have published data on the allele frequencies of the *FGF4L2* allele in Dachshunds, ranging from 88.00% to 99.00% [9,11,22,26], with individual studies reporting values of 88.00% [9], 91.79% [26], 95.00% [22], and 99.00% [11]. The overall allele frequency observed in the present study of 96.51% falls within this previously described range.

To our knowledge, the Veterinary Genetics Laboratory at the University of California, Davis, provides the largest available dataset on *FGF4L2* allele frequencies in Dachshunds. Based on aggregated genetic test results, the laboratory reports a breed-level allele frequency of approximately 91.79% [26]. While these data are derived from a large diagnostic testing database rather than a defined research cohort, they offer valuable insight into the allele’s overall distribution within the breed. The allele frequency observed in the present study is slightly higher but falls within a comparable range.

Furthermore, a comprehensive review reported an *FGF4L2* retrogene insertion allele frequency of 97.00% in 509 Dachshunds [17], derived from aggregated breed-level data across multiple studies. Although these data do not represent a single defined cohort, the reported frequency closely aligns with the overall allele frequency observed in the present study.

In addition, a study of 697 Dachshunds from Swiss and UK/US populations reported an allele frequency of 95.00%, providing further evidence for high *FGF4L2* allele frequencies in Dachshunds [22]. Although these data were derived from a combined reference population, the frequency closely resembles the overall allele frequency of 96.51% observed in the present study.

In contrast, Sullivan et al. (2025) reported an *FGF4L2* allele frequency of 88.00% in 407 Dachshunds registered with Scandinavian kennel clubs [9]. As inclusion required both radiographic spinal assessment and genotyping, this cohort represents a specifically screened breeding population. This may partly explain the slight difference compared to the overall allele frequency observed in the present study. A geographical component may also contribute to the observed variation, as Dickinson et al. (2020) reported differing allele frequencies in Dachshund populations from the USA/UK (98.00%) and Switzerland (94.00%) [17].

The highest allele frequency of the *FGF4L2* retrogene, at 99.00%, was found in Batcher et al.’s (2019) cross-breed study with 221 Dachshunds, in which they aimed to describe the association between the phenotype and the two *FGF4* retrogenes (*FGF4L1* and *FGF4L2*), as well as the risk of developing intervertebral disc extrusion (IVDE) [11]. In comparison to the overall allele frequency of our study (96.51%), where all nine Dachshund varieties were combined, Batcher’s study also combined all nine varieties and treated them as one breed, but based on weight, all except two Dachshunds were miniature size. Looking at our data and just using the miniature sizes, the allele frequency also exhibits a minimal increase to 97.85%. This is similar to the 99.00% allele frequency of the Batcher study. This suggests that differences in the composition of the study population may influence reported allele frequencies. Otherwise, subtle differences can be observed in the allele frequency of miniature Dachshunds categorised by hair type (99.07%, 98.18%, 96.30%) in our data. One possible explanation for this could be the small sample size per subgroup (*n* = 53–54), and thus, random genetic differences. Alternatively, it could be an indication that the nine varieties should not be combined, but rather split into subgroups and analysed separately. 

In preceding studies, in which the hair type groups, but not the size, are differentiated, the allele frequencies interestingly differ greatly [27]. A substantially lower *FGF4L2* allele frequency of 74.00% has been reported specifically in wire-haired Dachshunds [27], which aligns with the comparatively lower frequency observed in wire-haired Dachshunds in the present study. In contrast, the same study reported that the insertion allele was nearly fixed in smooth-haired and long-haired Dachshunds [27], indicating pronounced variation between coat types. Although that investigation evaluated the relationship between coat type and the incidence of disc herniation, differentiation by size category was not performed.

However, most previous investigations did not differentiate between both coat and size subgroups, potentially masking underlying heterogeneity within the breed.

Nevertheless, a comprehensive genetic study involving over one million dogs found that the *FGF4L2* allele frequencies are 99.50% in miniature long-haired (*n* = 213) and 95.00% in miniature short-haired Dachshunds (*n* = 583) [20]. The frequency seen in miniature long-haired Dachshunds was comparable to that of the current study (98.18%), whereas the miniature short-haired subgroup in the German DTK population exhibited a slightly higher allele frequency (99.07%). These findings further support the notion that allele frequencies may vary between Dachshund populations and breeding structures.

Particularly noteworthy was the lower *FGF4L2* allele frequency observed in wire-haired standard Dachshunds (89.09%) in the present study. Comparable observations have been reported in Bruun’s study [27]. One possible explanation for the slightly higher genetic variation in the wire-haired Dachshund group might be that Schnauzer and Terrier breeds have been bred into them more frequently to create the hair structure [17], resulting in a more frequent refreshment of their genetic pool. This would explain why their subpopulation is not genetically as fixed as the others. Furthermore, the historical introduction of Terrier-type breeds may have influenced allele frequencies in wire-haired Dachshunds, as the *FGF4L2* insertion has been reported to occur at low frequencies in Terrier breeds [17].

This observation is consistent with the reported association between *FGF4L2* and intervertebral disc extrusion [10], and is emphasised by the fact that clinically apparent IVDD has the lowest reported prevalence in standard wire-haired Dachshunds, at 7.1% [7]. In contrast, Packer et al. reported the highest prevalence of clinically reported IVDD in standard smooth-haired Dachshunds, at 24.4% [7]. This is interesting because, in the present study, this subgroup had the highest number of heterozygous genotypes (*N/FGF4L2*) and an allele frequency of 91.67%. While allele frequencies in our dataset of long-haired Dachshunds are almost fixed (98.11–100.00%), the DachsLife study observed a 12.6% prevalence of clinically apparent signs of IVDD in standard long-haired Dachshunds [7]. Recently, Hopper et al. [21] also reported better vertebral column health in both wire-haired and long-haired Dachshunds compared with smooth-haired Dachshunds. While the lower *FGF4L2* allele frequency observed in standard wire-haired Dachshunds may contribute to this finding, the similarly favourable vertebral column health reported for long-haired Dachshunds despite near-complete fixation of *FGF4L2* suggests that variation in *FGF4L2* allele frequency alone may not fully account for these differences.

These observations suggest that the manifestation of clinically apparent IVDE is likely influenced by multiple factors beyond the presence of the *FGF4L2* insertion alone. Additional genetic and non-genetic factors may contribute to the observed differences between Dachshund subgroups. Furthermore, comparisons should be interpreted cautiously, as genotype and clinical data were obtained from different study populations.

Although the lower *FGF4L2* allele frequency observed in standard wire-haired Dachshunds is consistent with our a priori hypothesis based on the previously reported lower prevalence of clinically apparent IVDD in this variety, the similarly favourable vertebral column health reported for long-haired Dachshunds despite near-complete fixation of *FGF4L2* indicates that variation in *FGF4L2* allele frequency alone is unlikely to fully explain the observed differences between Dachshund coat varieties. Further studies in independent Dachshund cohorts, integrating detailed clinical phenotyping with additional genetic and non-genetic factors, are warranted to better understand the multifactorial basis of vertebral column health and clinically apparent IVDE.

Nevertheless, these observations do not preclude a quantitative effect of the *FGF4L2* insertion itself. In this context, imaging data from young, asymptomatic Nova Scotia Duck Tolling Retrievers (NSDTR), a breed that is useful to study since it does not typically carry the *FGF4L1* retrogene, supports the idea of a dose-dependent relationship between the *FGF4L2* genotype and disc calcification [28]. In the NSDTR study, no intervertebral calcifications were found in homozygous wild-type individuals, whereas calcifications were found in almost all heterozygous individuals except one. Furthermore, heterozygotes had approximately half the number of calcifications of homozygous CDDY carriers, suggesting a gene dose effect in this breed [28].

In 2024, Sullivan then examined the number of *FGF4L2* insertion copies in Dachshunds from the Norwegian and Finnish kennel clubs and investigated the correlation between this number and radiographic disc calcification [9]. Interestingly, differences in radiographic disc calcification scores were observed between dogs carrying zero and two copies of the insertion, whereas individuals with one copy showed calcification scores more similar to those of wild-type dogs than to dogs homozygous for the *FGF4L2* insertion [9].

The differing conclusions of these studies may partly reflect variations in study design and genotype distribution. While the Sullivan study included 407 Dachshunds, only seven dogs were wild-type, compared with 80 heterozygous and 320 homozygous *FGF4L2* carriers, whereas the NSDTR study, despite including only 22 dogs, was based on more balanced genotype groups.

Additionally, breed-specific genetic and population structure may contribute to the observed discrepancies, as the biological impact of the *FGF4L2* insertion could differ between breeds.

However, comparisons between the results of these studies should be interpreted cautiously, as disc calcifications were assessed using different imaging modalities (computed tomography versus radiography).

The possible clinical relevance of these observations becomes more obvious when considering previous studies linking disc calcification to clinically apparent IVDE. In Dachshunds, increasing numbers of radiographically detectable calcified discs have been associated with a higher risk of disc extrusion and clinically apparent IVDE [29,30].

Consequently, the observed association between *FGF4L2* genotype and disc calcification may not be limited to imaging findings alone. Taken together, these studies are consistent with the hypothesis that increasing numbers of *FGF4L2* insertion copies may also be associated with an increased risk of clinically apparent IVDE, although direct evidence linking genotype, disc degeneration, and clinical outcome within the same populations remains limited.

In Dachshunds, these findings are consistent with the possibility that heterozygous dogs exhibit an intermediate disc calcification phenotype between wild-type and homozygous *FGF4L2* carriers, although the relationship between disc calcification and the development of clinically apparent IVDE is not yet fully understood. This observation may be of interest in the context of breeding strategies aimed at reducing the frequency of the *FGF4L2* insertion while preserving breed characteristics.

Previous studies have suggested that controlled breeding strategies targeting heterozygous individuals may help to gradually reduce disease risk while maintaining breed-defining traits [9].

The persistence of both heterozygous and wild-type genotypes, particularly in standard wire-haired and standard smooth-haired Dachshunds, indicates that genetic variation at the *FGF4L2* locus remains within parts of the DTK-registered Dachshund population. Notably, homozygous wild-type individuals were identified exclusively in the standard wire-haired subgroup. Together, these findings suggest that opportunities remain for future breeding approaches aimed at decreasing the *FGF4L2* allele frequency.

In this context, the potential role of exchange between varieties and subpopulations warrants further consideration.

However, any effort to reduce *FGF4L2* allele frequencies should be balanced against the need to preserve genetic diversity within the breed. Given the high prevalence of the insertion allele, relying on a few wild-type individuals or applying excessive selection pressure may increase the risk of genetic bottlenecks and inbreeding.

As a result, the potential benefits of incorporating wild-type alleles therefore need to be considered alongside the long-term preservation of genetic diversity across the different Dachshund varieties and subpopulations.

At the same time, a more comprehensive understanding of the multifactorial basis of clinically apparent IVDE and vertebral column health will be important for optimising future breeding strategies. Nevertheless, given the well-established association of *FGF4L2* with premature intervertebral disc degeneration and clinically apparent IVDE, consideration of this locus remains a reasonable first step within a broader, evidence-based breeding approach.

Returning to the main problem and considering the well-established association between the *FGF4L2* insertion and premature degeneration of the intervertebral disc, resulting in an increased risk of clinically apparent sequelae, the high allele frequency observed in this study provides important context for future discussions regarding breeding strategies and vertebral column health in the German Dachshunds registered with the DTK.

Further prospective controlled studies are needed to better understand the factors influencing the development of clinically apparent IVDE and the interaction between genetic, environmental, and lifestyle-related influences. In the long term, carefully managed breeding approaches that balance a reduction in *FGF4L2* allele frequencies with the preservation of genetic diversity may contribute to improving vertebral column health within the breed.

When interpreting the findings of this study, several limitations should be given consideration. The study population was derived from registered breeding dogs of the DTK databank within a national context and therefore does not represent the entire German Dachshund population. The population is not a completely random cross-section of the breed because the dogs included may be selected based on breeding programmes, and it may reflect previous selection based on health considerations, resulting in selection bias.

Another limitation of the present study is the sample size within individual breed varieties. Based on the expected allele frequency distribution, larger cohorts would have been required to achieve narrower confidence intervals for subgroup-specific allele frequency estimates. Due to financial constraints, the target sample size could not be reached, and the resulting estimates should therefore be interpreted with appropriate caution. Nevertheless, the observed differences between subpopulations provide valuable preliminary data for future investigations and breeding considerations.

Although no significant deviations from Hardy–Weinberg equilibrium were detected in either the overall study population or the individual Dachshund subgroups, the results should be interpreted in the context of the structured DTK breeding programme. As mating is controlled rather than random, Hardy–Weinberg equilibrium provides only limited information on the underlying population structure.

## 5. Conclusions

This study provides the first subgroup-specific baseline data on the frequency of the *FGF4L2* retrogene, and these data may support the interpretation of future genetic screening results in the DTK Dachshund population. This study revealed meaningful variation across coat and size types. While the insertion allele was still very common overall, there was still detectable genetic variation at this locus in some subpopulations, especially standard wire-haired Dachshunds. These findings both demonstrate that grouping Dachshund varieties into a single breed population may obscure important subgroup-specific differences and highlight the importance of considering genetically distinct subpopulations within the breed, including coat and size varieties, separately in future genetic studies.

Additionally, our findings cannot fully explain the reported differences in the prevalence of clinically apparent IVDE and vertebral column health between Dachshund coat varieties, indicating that factors other than *FGF4L2* also contribute to these differences and underlining the multifactorial nature of IVDE. Nonetheless, *FGF4L2* remains the best-characterised genetic risk factor identified to date, and the marked variation in its allele frequency between varieties makes it a concrete and immediately actionable target for breeding strategies. The *FGF4L2* genotype should therefore be integrated into existing breeding and health-screening programmes as one component of a broader, evidence-based strategy. Continued investigation of the additional genetic and non-genetic factors influencing the development of clinically apparent IVDE will be required to improve long-term vertebral column health in Dachshunds.

## Figures and Tables

**Table 1:** Genotype distribution of *FGF4L2* in 488 Dachshunds.

Genotype	Count	Percentage
*FGF4L2/FGF4L2*	456	93.44%
*N/FGF4L2*	30	6.15%
*N/N*	2	0.41%

**Table 2:** *FGF4L2* genotype and allele frequencies across Dachshund coat-type and size varieties.

Coat Type	Size	*n*	*FGF4L2/FGF4L2* *n* (%)	*N/FGF4L2* *n* (%)	*N/N* *n* (%)	Allele Frequency (%)	95% Wilson CI (%)
Smooth	Standard	54	45 (83.33)	9 (16.67)	0 (0.00)	91.67	84.92–95.55
Smooth	Miniature	54	53 (98.15)	1 (1.85)	0 (0.00)	99.07	94.94–99.84
Smooth	Rabbit	53	51 (96.23)	2 (3.77)	0 (0.00)	98.11	93.38–99.48
Long	Standard	54	54 (100.00)	0 (0.00)	0 (0.00)	100.00	96.57–100.00
Long	Miniature	55	53 (96.36)	2 (3.64)	0 (0.00)	98.18	93.61–99.50
Long	Rabbit	54	52 (96.30)	2 (3.70)	0 (0.00)	98.15	93.50–99.49
Wire	Standard	55	45 (81.82)	8 (14.55)	2 (3.64)	89.09	81.90–93.65
Wire	Miniature	54	50 (92.59)	4 (7.41)	0 (0.00)	96.30	90.86–98.55
Wire	Rabbit	55	53 (96.36)	2 (3.64)	0 (0.00)	98.18	93.61–99.50

## Data Availability

The data presented in this study are available from the corresponding author upon reasonable request.

## Supplementary Materials

The following supporting information can be downloaded at: https://www.mdpi.com/article/10.3390/genes17080969/s1, Table S1. Results of the exact Hardy–Weinberg equilibrium tests for each Dachshund variety.; Figure S1. Genotype distribution across the nine Dachshund varieties.

## Abbreviations

The following abbreviations are used in this manuscript:

IVD	Intervertebral disc
IVDD	Intervertebral disc disease
IVDE	Intervertebral disc extrusion
CDDY	Chondrodystrophy
CDPA	Chondrodysplasia
FGF4L1	Fibroblast growth factor 4 retrogene on chromosome 18
FGF4L2	Fibroblast growth factor 4 retrogene on chromosome 12
DTK	Deutscher Teckelklub 1888 e.V.
DNA	Deoxyribonucleic acid
PCR	Polymerase chain reaction
