# Supplementary material for: Genotypic Distribution of FGF4L2 in DTK-Registered Dachshunds in Germany: A Pilot Study
Source: Genes (Basel). 2026 Aug 19;17(8):969. doi: 10.3390/genes17080969 (PMC13512071; doi:10.3390/genes17080969)

Supplementary Table S1. Results of the exact Hardy–Weinberg equilibrium tests for each Dachshund variety.

Dachshund variety	FGF4L2/FGF4L2	N/FGF4L2	N/N	Exact HWE *p*-value
**Smooth Standard**	45	9	0	1.000
**Smooth Miniature**	53	1	0	1.000
**Smooth Rabbit**	51	2	0	1.000
**Long Standard**	54	0	0	1.000
**Long Miniature**	53	2	0	1.000
**Long Rabbit**	52	2	0	1.000
**Wire Standard**	45	8	2	0.106
**Wire Miniature**	50	4	0	1.000
**Wire Rabbit**	53	2	0	1.000

Supplementary Figure S1. Genotype distribution across the nine Dachshund varieties. Stacked bar chart showing the percentage distribution of FGF4L2 genotypes (FGF4L2/FGF4L2, N/FGF4L2 and N/N) within each of the nine Dachshund varieties.

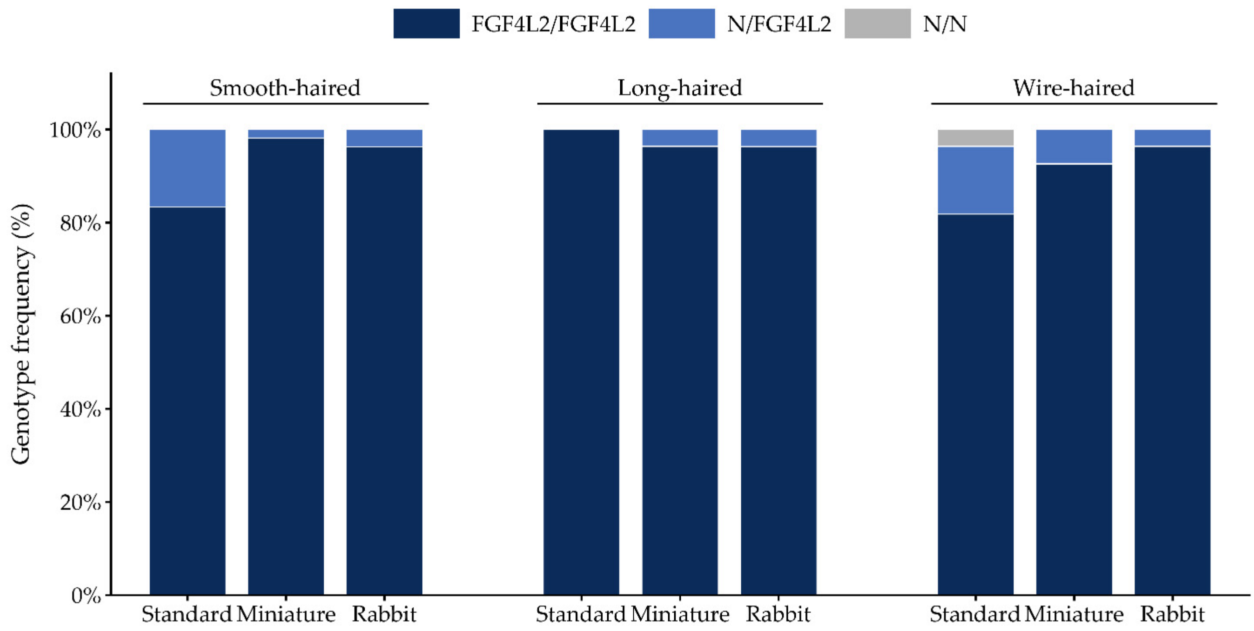

Supplement: Supplementary file 1 [file genes-17-00969-s001.zip › genes-4440134-supplementary.pdf]
